# *Lactococcus lactis* subsp. *Cremoris* C60 restores T Cell Population in Small Intestinal Lamina Propria in Aged Interleukin-18 Deficient Mice

**DOI:** 10.3390/nu12113287

**Published:** 2020-10-27

**Authors:** Suguru Saito, Nanae Kakizaki, Alato Okuno, Toshio Maekawa, Noriko M. Tsuji

**Affiliations:** 1 Division of Cellular and Molecular Engineering, Department of Life Technology and Science, National Institute of Advanced Industrial Science and Technology (AIST), Tsukuba, Ibaraki 3058560, Japan; nanatocchi1128-kakizaki@aist.go.jp (N.K.); okuno@ifoodmed.jp (A.O.); toshio.maekawa@aist.go.jp (T.M.); 2 iFoodMed Inc., Tsuchiura, Ibaraki 3000873, Japan

**Keywords:** probiotics, small intestine, intestinal immunity, Interleukin-18, IFN-γ+CD4+ T (Th1) cells, dendritic cells, lamina propria (LP), Peyer’s patch (PP)

## Abstract

Lactic acid bacteria (LAB), a major commensal bacterium in the small intestine, are well known beneficial bacteria which promote establishment of gut-centric immunity, such as anti-inflammation and anti-infection. In this report, we show that a LAB strain *Lactococcus lactis* subsp. *Cremoris* C60 possess an ability to activate antigen presenting cells, such as dendritic cells (DCs), and intestinal T cells which possibly support to maintain healthy intestinal immunological environment in aging process. We found that CD4+ T cells in the small intestine are dramatically decreased in aged Interleukin-18 knock out (IL-18KO) mice, associated with the impairment of IFN-γ production in the CD4+ T cells, especially in small intestinal lamina propria (LP). Surprisingly, heat killed-C60 (HK-C60) diet completely recovered the CD4+ T cells population and activity in SI-LP and over activated the population in Peyer’s patches (PPs) of IL-18KO mice. The HK-C60 diet was effective approach not only to restore the number of cells, but also to recover IFN-γ production in the CD4+ T cell population in the small intestine of IL-18-deficient mice. As a possible cause in the age-associated impairment of CD4+ T cells activity in IL-18KO mice, we found that the immunological activity was downregulated in the IL-18-deficient DCs. The cytokines production and cellular activation markers expression were downregulated in the IL-18-deficient bone marrow derived dendritic cells (BMDCs) at the basal level, however, both activities were highly upregulated in HK-C60 stimulation as compared to those of WT cells. Antigen uptake was also attenuated in the IL-18-deficient BMDCs, and it was significantly enhanced in the cells as compared to WT cells in HK-60 stimulation. An in vitro antigen presentation assay showed that IFN-γ production in the CD4+ T cells was significantly enhanced in the culture of IL-18-deficient BMDCs compared with WT cells in the presence of HK-C60. Thus, we conclude that HK-C60 diet possesses an ability to restore T cells impairment in the small intestine of IL-18-deficient environment. In addition, the positive effect is based on the immunological modification of DCs function which directory influences into the promotion of effector CD4+ T cells generation in the small intestine.

## 1. Introduction

Lactic acid bacteria (LAB) are recognized as one of the beneficial bacterial strains in our body by contributing into the establishment of healthy intestinal immune environment [1]. LAB have traditionally been identified from fermented foods, and they are one of the major strains of commensal bacterium in the small intestine as colonizing trillion order [2]. Some LAB strains possess a unique effect in the enhancement and modification of immune responses in both innate and adaptive immunity [3,4]. As following a general strategy of host immune response against microbes, LAB are firstly recognized by innate immune cells such as macrophages (Mφ) and dendritic cells (DCs). The exposure with LAB enhances innate immune cells activity, then the subsequent immunological responses, such as T cells activation in adaptive immunity, are driven to showing various immunological responses. However, this is a non-inflammatory response in the current understanding of immunology [5,6]. In the recognition step of LAB in the immune cells, some reports showed that innate immune receptors, such as Toll-like receptors (TLRs), contribute to the capture of LAB and generation of downstream signaling which finally induces cytokine production and cellular activation in the cells [7,8].

We previously reported that various immune responses were triggered/regulated by LAB in both murine and human cell-based model. We showed that *Tetragenococcus halophilus* KK221 established a protective immune response in dextran sulfate sodium (DSS)-induced colitis, which a murine experimental model of ulcerative colitis [9]. As an underlying mechanism, we found that double-strand RNA (dsRNA) is abundantly produced from KK221 sensed through endosomal TLR3, and this response induced interferon-beta (IFN-β) production in the intestinal DCs. In addition, we also reported that *Pediococcus acidilactici* K15 promoted IFN-γ production from CD4+ T cells rather than IL-4 in the ex vivo system using human peripheral blood mononuclear cells (PBMCs) originated myeloid DCs (mDCs) and naïve CD4+ T cells [10]. Thus, LAB, despite their non-pathogenic character, induce a dynamic response in the host immunity.

Interleukin (IL)-18 was identified as an important cytokine in the regulation of T cells activity. IL-18 was characterized as having a similar function as IL-12 in the upregulation of IFN-γ production induced by T cell receptor (TCR) stimulation in CD4+ T cells [11]. IL-18 is secreted from various innate immune cells such as macrophages and DCs, and the secretion helps activation and maintenance of T cell population [12,13,14]. Due to their character in the immune system, IL-18 deficiency shows critical influence in T cells activity. For instance, IL-18 deficient mice were susceptible to viral infection [15]. IL-18 production is regulated by inflammasome dependent manner as same as IL-1β. Nod-like receptors (NLRs) are important sensor to induce the signal of which the directory promotes inflammasome assembly and the activation of caspase-1 in both IL-18 and IL-1β production [16]. IL-18 deficiency promotes a loss of resistance against pathogens, such as tuberculosis, infected in macrophages [17]. In the immune response of DCs, IL-18 is also important cytokine to upregulate their activity in a self-activation manner through IL-18 receptor (IL-18R) [18,19]. Innate immune cells are working as important players for establishing initial defense against infection and tissue damage by exposing with both exogenous and endogenous stimuli in mucosa layer [20,21]. IL-18 deficiency abrogates the innate immune response in mucosa, so that the subsequent immunological response, such as T cell-based adaptive immunity, is attenuated by the cytokine’s defect [22]. In addition, IL-18 contributes to the maintenance of epithelial homeostasis in the gut, which also influences into the immunological responses [23]. Lamina propria (LP) in the small intestine has a similar character as other mucosa layers, means which macrophages and DCs are constantly working for the maintenance of the innate defense and regulation of adaptive immunity in the tissue. Although IL-18 deficiency had never reported as a critical factor in the establishing of T cell based adaptive immunity in small intestine yet, we suspected that the deficiency of IL-18 might be a cause of the functional failure of T cells environment in the organ.

In this report, we show that *Lactococcus lactis* subsp. *Cremoris* C60 recovers the T cell population and function, which are down regulated in small intestine of aged IL-18 KO mice. Aged IL-18 KO mice showed a severe decreasing of CD3+ T cell population in the small intestinal lamina propria (SI-LP). As the most severely influenced subset in the CD3+ lineage, we found CD4+ T cells decreasing in the SI-LP of aged IL-18KO mice. The T cells decreasing was an age-relatedly accelerated event in the SI-LP of IL-18KO mice because the dramatic CD3+ T cells decreasing was observed in aged mice rather than young mice in the strain. Along with the decreasing of CD4+ T cell population, IFN-γ producing CD4+ T (Th1) cells were also significantly decreased in SI-LP of aged IL-18KO mice as compared to that of WT mice. The heat killed (HK)-C60 diet dramatically restored the T cell population in SI-LP of aged IL-18KO mice. In addition, the diet recovered small intestinal CD4+ T cells activities which were characterized as upregulation of IFN-γ production and cell proliferation in the aged IL-18 KO mice. Furthermore, the TCR-dependent reactivity of CD4+ T cells which was impaired in IL-18KO mice was enhanced by HK-C60 stimulation.

As one of the possible mechanisms in the recovery of CD4+ T cells activity, we found that HK-C60 enhanced the immunological function of IL-18-deficient DCs which directly influences the generation and maintenance of effector CD4+ T cells including Th1 cells. HK-C60 stimulation over promoted the cellular activity in IL-18-deficient bone marrow derived dendritic cells (BMDCs). The cytokines production and surface molecules expression were slightly downregulated in IL-18-deficient BMDCs in the basal status, however, both of those were significantly upregulated in HK-C60 stimulated IL-18-deficient BMDCs as compared to that of WT cells. The BMDCs activities which tightly correlated with antigen-specific CD4+ T cells generation, such as antigen uptake and antigen presentation, were both enhanced in IL-18-deficient BMDCs when the cells were exposed with HK-C60. As a consequence, the generation of Th1 cells was significantly promoted by IL-18-deficient BMDCs in the presence of HK-C60 as compared to that of WT cells.

Taken together, C60 has an ability to argument CD4+ T cells related immunological environment which is selectively suppressed by IL-18-deficiency in the small intestine. This finding supports a possibility of the usage of C60 in a probiotic approach for the maintenance or promotion of healthy gut immunity.

## 2. Materials and Methods

### 2.1. Mice

BALB/c (WT) mice were purchased from Japan Clea (Tokyo, Japan). IL-18KO (*Il-18^−/−^*, BALB/c background) mice were provided from Yeal University (Kiyoshi Takeda). The WT and IL-18KO were initially crossed to gain hetero IL-18KO (*Il-18^+/−^*) mice. The hetero IL-18KO mice were further crossed to gain WT *(Il-18^+/+^*) and IL-18KO mice. This crossing was performed at least for 2 generations. The littermate of WT and IL-18KO were used for experiments. DO11.10 mice were provided from the University of Washington (Dr. Kenneth Murphy). All mice were bred and maintained in specific pathogen free (SPF) conditions with 12 h day/night cycles and were allowed free access to food and water. Gender-matched adult mice (10–12 weeks for young group and over 10 months for aged group) were used for each experiment. To investigate the effect of HK-LAB diet in intestinal immunological environment, the aged WT and IL-18KO mice were fed with AIN-93G pellet (control; Oriental yeast, Tokyo, Japan) or HK-C60 kneaded pellet (AIN-93G based). In the diet, the mice were firstly fed with control pellet for 2 weeks for habituation followed by HK-C60 kneaded or control pellet for 2 weeks. Some mice used as the cell source of PP cells or BM cells were fed with normal diet (CE-2; Japan Clea). All the mice have been bred in the facility since 2012. All experiments protocols were reviewed and approved by the Animal Welfare Committee of AIST (protocol No.109).

### 2.2. Lactic Acid Bacteria Culture

*Lactococcus lactis* subsp. *Cremoris* C60 was cultured by following method described in a previous report [24]. Briefly, the bacteria were cultured in MRS broth (BD Difco^TM^, BD Bioscience, Franklin Lakes, NJ, USA) at 30 ℃ for 24 h. The bacterial colony forming unit (CFU/mL) was calculated in all cultures. For HK-C60 preparation, the bacteria were autoclaved at 95 ℃ for 10 min, then the bacterial cells were precipitated by centrifugation at 5000× *g* for 10 min. After being washed with saline (0.9% NaCl), the pellet was finally suspended in saline. The suspension was used as HK-C60 for each experiment.

### 2.3. Reagents and Antibodies

Phorbol 12-myristate 13-acetate (PMA), ionomycin and ovalbumin (OVA) were purchased from Sigma Aldrich (St. Louis, MO, USA). Collagenase-D, Collagenase type-I, DNase, and Alexa 488-conjugated OVA were purchased from Thermo Fisher Scientific (Waltham, MA, USA). A Cytofix/Cytoperm kit with GolgiStop^TM^ was purchased from BD Bioscience (Franklin Lakes, NJ, USA). Recombinant murine interleukin-2 (rmIL-2) were purchased from Peprotech (Rocky Hill, NJ, USA). Recombinant murine granulocyte macrophage-colony stimulating factor (rmGM-CSF), Fluorescein isothiocyanate (FITC) or phycoerythrin/cyanin 7 (PE/Cy7)-conjugated anti-CD45 (30-F11), FITC or allophycocyanin (APC)-conjugated anti-CD3ε (17A2), allophycocyanin/cyanin 7 (APC/Cy7) or brilliant violet 510 (BV510)-conjugated anti-CD4 (GK1.5), Pacific blue or PE/Cy7-conjugated anti-CD8α (53-6.7), BV421-conjugated anti-CD11c (N418), FITC-conjugated anti-CD80 (16-10A1), phycoerythrin (PE)-conjugated anti-CD86 (GL-1), APC-conjugated anti-MHC-II (I-A/I-E; M5/114.15.2), PE or PE/Cy7-conjugated anti-interferon gamma (IFN-γ) (XMG1.2), PE-conjugated anti-IL-12/IL-23p40 (C11.5), APC-conjugated anti-IL-6 (MP5-20F3), FITC-conjugated anti-TNF-α (MP6-XT22), purified anti-CD16/CD32 (2.4G2), Ultra-LEAF^TM^ purified anti-CD3 (17A2), Ultra-LEAF^TM^ purified anti-CD28 purified (37.51) and CFSE Cell Division Tracker Kit were all purchased from Biolegend (San Diego, CA, USA). The isotype control antibodies were purchased from the same company.

### 2.4. Small Intestinal-Lamina Propria (SI-LP) Cells Isolation

SI-LP cells were isolated by following a protocol described in a previous report with minor modification [25]. Briefly, the small intestine was extracted from mouse, then was washed in phosphate-buffered saline (PBS). The intestine was opened by cutting with scissors and washed inside well with PBS. The intestine was cut to small pieces (5–10 mm) and incubated in a washing buffer (PBS containing 10% Fetal bovine serum (FBS), 10 mM ethylenediaminetetraacetic acid (EDTA), 20 mM 4-(2-hydroxyethyl)-1-piperazineethanesulfonic acid (HEPES), 100 mg/mL penicillin, 100 mg/mL streptomycin, 2 mM sodium pyruvate) at 37 °C for 30 min with stirring. After incubation, the intestine pieces were washed in PBS several times, then were further cut to small pieces as much as possible (1–2 mm) in cell digestion buffer (RPMI 1640 medium supplemented with 10% FBS, 20 mM HEPES, 100 mg/mL penicillin, 100 mg/mL streptomycin, 1 mg/mL collagenase-D, 50 μg/mL DNase I). The sample was incubated at 37 °C for 30 min with stirring. After digestion, the sample was filtered through on a 70 μm cell strainer and undigested tissue pieces were mechanically crushed on the cell strainer. The isolated cells were washed with cell culture medium (RPMI 1640 supplemented with 10% FBS, 50 μM 2-mercaptoethanol (2-ME), 10 mM HEPES, 100 mg/mL penicillin, 100 mg/mL streptomycin), then were again filtered through on a 40 μm cell strainer. After being washed with cell culture medium, the cells were finally collected by centrifugation at 300× *g* for 5 min. The precipitated cells were used as SI-LP isolated cells.

### 2.5. Peyer’s Patch (PP) Cells Isolation

PPs were extracted from small intestine, then were briefly washed with PBS. The PPs were incubated in epithelial dissociation buffer (PBS containing 10% FBS, 100 mg/mL penicillin, 100 mg/mL streptomycin, 20 mM EDTA and 10 mM dithiothreitol (DTT)) at 37 °C for 20 min with staring. The PPs were collected by decantation onto a 70-μm cell strainer, then briefly washed with cell culture medium. The PPs were transferred to digestion buffer (RPMI1640 supplemented with 10% FBS, 100 mg/mL penicillin, 100 mg/mL streptomycin, 1 mg/mL collagenase type-I, 50 μg/mL DNase) and incubated at 37 °C for 30 min with staring. After digestion, the PPs were collected by decantation onto a 70-μm cell strainer, then crushed on the strainer in cell culture medium. The cells were washed with cell culture medium, then the sample was filtered through a 40 μm cell strainer again. After being washed with cell culture medium, the cells were finally collected by centrifugation at 300× *g* for 5 min. The precipitated cells were used as PP isolated cells.

### 2.6. Splenocytes Preparation

Splenocytes preparation was performed by following a protocol described in a previous report [26]. Briefly, the extracted spleen was mechanically crushed on a 70 μm cell strainer with cell culture medium. The cells were washed with cell culture medium, then the cells were precipitated by centrifugation at 300× *g* for 5 min. The cell pellet was re-suspended in 1× RBC lysis buffer and was incubated at RT for 10 min. The cell suspension was neutralized with cell culture medium to stop the reaction, and the cells were precipitated by centrifugation at 300× *g* for 5 min. The cells were washed with cell culture medium, then were finally collected by centrifugation at 300× *g* for 5min. The precipitated cells were used as splenocytes. Splenic CD4+ T cells were isolated from splenocytes prepared from spleen of DO11.10 mice by using CD4+ T Cell Isolation Kit (Miltenyi Biotec, Bergisch Gladbach, Germany). All procedure was performed by following the product manual. The purity of isolated CD4+ T cells was analyzed by flow cytometry in each isolation. The sample with over 90% of CD3+CD4+ population was used for experiment.

### 2.7. Flow Cytometry

To characterize immunological phenotype of T cells and DCs, the samples were analyzed by a flow cytometer (FACS Aria I; BD Biosciences) with the fluorochrome-conjugated monoclonal antibodies described in reagents and antibodies. In the sample preparation, the cells were firstly treated with FcR blocker (anti-CD16/CD32) at 4 °C for 10 min. After being washed with PBS/2% FBS, the cell surface markers were stained with the antibody in PBS/2% FBS at 4 °C for 30 min. For intracellular staining, the cells were treated with Cytofix/CytoPerm Kit with GolgiStop^TM^ (BD Biosciences) by following the product manual. All staining was performed by following the Ab combination listed in Supplementary Table S1. The dead cells were excluded by forward scatter, side scatter and 7-Amino-Actinomycin D (7-AAD) in each analysis. All data were analyzed by BD FACS Diva (BD bioscience) or FlowJo (BD bioscience).

### 2.8. In Vitro and Ex Vivo Stimulation of PP Isolated Cells

PP isolated cells (1.0 × 10^7^/mL) were seeded on anti-CD3 mAb (10 μg/mL) pre-coated 96 well plate in cell culture medium containing anti-CD28 mAb (2 μg/mL) for both in vitro and ex vivo stimulation. Some cells were pre-stained with 5-(and 6)-Carboxyfluorescein diacetate succinimidyl ester (CFSE) prior to seeding on the plate for in vitro stimulation. For ex vivo stimulation, which mimic the natural internal environment of PP, the culture was further treated with HK-C60 (1.0 × 10^8^ CFU/mL) or vehicle control. The culture was incubated at 37 °C for 48 h. For intracellular cytokine detection, the cells were re-stimulated with PMA (500 ng/mL) and ionomycin (1 μg/mL) in the presence of GolgiStop^TM^ at the last 5 h. Finally, the all the samples were analyzed by flow cytometry.

### 2.9. Bone Marrow Derived Dendritic Cells (BMDCs) Preparation

BMDCs were prepared by following protocols described in previous reports with modification [27,28]. Briefly, the BM cells were flushed out from the tibia and femur onto a 70 μm of cell strainer by using a 10 mL syringe with a 27G needle containing cell culture medium. The cell suspension were filtered through a 70 μm cell strainer and the cells were washed with cell culture medium. The cells were precipitated by centrifugation at 300× *g* for 5 min, and the cells were re-suspended in 1×RBC lysis buffer. The cells were incubated at RT for 10 min, then the sample was neutralized with cell culture medium to stop the reaction. The cells were washed with cell culture medium, and the cells were finally collected by centrifugation at 300× *g* for 5 min. The precipitated cells were used as BM cells. BM cells (3.0 × 10^5^/mL) were seeded on 6 well plate with DC culture medium (cell culture medium supplemented with 20 ng/mL of rmGM-CSF), and the plate was incubated at 37 °C. At day 3 and 6, the half volume of medium was replaced to fresh DC culture medium. At day 8, the cells were harvested and CD11c+ population was enriched by using CD11c Micro Beads Ultra Pure (Miltenyi Biotec). All procedures were performed by following the product manual. The BMDCs quality was analyzed by flow cytometry in every culture. The sample with over 90% of CD11c+ and maintained CD80^low^, CD86^low^ and I-A/I-E^low^ in the CD11c+ population was used for experiment.

### 2.10. BMDCs Stimulation Assay

BMDCs (1.0 × 10^6^/mL) were seeded on 12 well plate or 96 well plate in cell culture medium for flow cytometry or ELISA respectively. For intracellular cytokine detection, the cells were stimulated with HK-C60 (5.0 × 10^7^ CFU/mL) in the presence of GolgiStop^TM^ at 37 °C for 6 h. For analysis of surface maker expression, the cells were stimulated with HK-C60 (5.0 × 10^7^ CFU/mL) at 37 °C for 24 h. The samples were analyzed by flow cytometry. For cytokine production assay by ELISA, the cells were stimulated with HK-C60 (5.0 × 10^7^ CFU/mL) or LPS (10 ng/mL) at 37 °C for 24 h. Some samples were treated with vehicle control in each assay. The cultured medium was harvested and stored at −80 °C until use.

### 2.11. Antigen Uptake Assay

BMDCs (1.0 × 10^6^/mL) were seeded on 12 well plate in cell culture medium. OVA-Alexa 488 (1 μg/mL) was added into the culture as antigen. The cultures were treated with HK-C60 (5.0 × 10^7^ CFU/mL) or vehicle control. The plate was incubated at 37 °C for 24 h, and the cells were harvested and analyzed by flow cytometry.

### 2.12. Antigen Presentation Assay

BMDCs (2.0 × 10^5^/mL) and DO11.10 CD4+ T cells (1.0 × 10^6^/mL) were seeded on 96 well plate in cell culture medium supplemented with rmIL-2 (10 ng/mL). OVA_323–339_ peptide (1 μg/mL) was added into the culture as antigen. Some cultures were treated with HK-C60 (1.0 × 10^7^ CFU/mL) or vehicle control. The culture was incubated at 37 °C for 72 h. The proliferated cells were re-stimulated with PMA (500 ng/mL) and ionomycin (1 μg/mL) in the presence of GolgiStop^TM^ at the last 5 h of culture. The sample was analyzed by flow cytometry.

### 2.13. Enzyme-Linked Immuno Sorbent Assay (ELISA)

The cytokines (IL-6, IL-12, and TNF-α) produced from stimulated BMDCs were measured by using Duo set ELISA kit (R&D systems, Minneapolis, MN, USA). The procedure was performed by following the product manual.

### 2.14. Statistics

A Mann–Whitney U test was used to analyze the data for significant differences. Values of *p* < 0.05, *p* < 0.01, and *p* < 0.001 were regarded as significant.

## 3. Results

### 3.1. HK-C60 Diet Restores T Cell Population Which Decreased in SI-LP of Aged IL-18KO Mice

**Figure 1:**
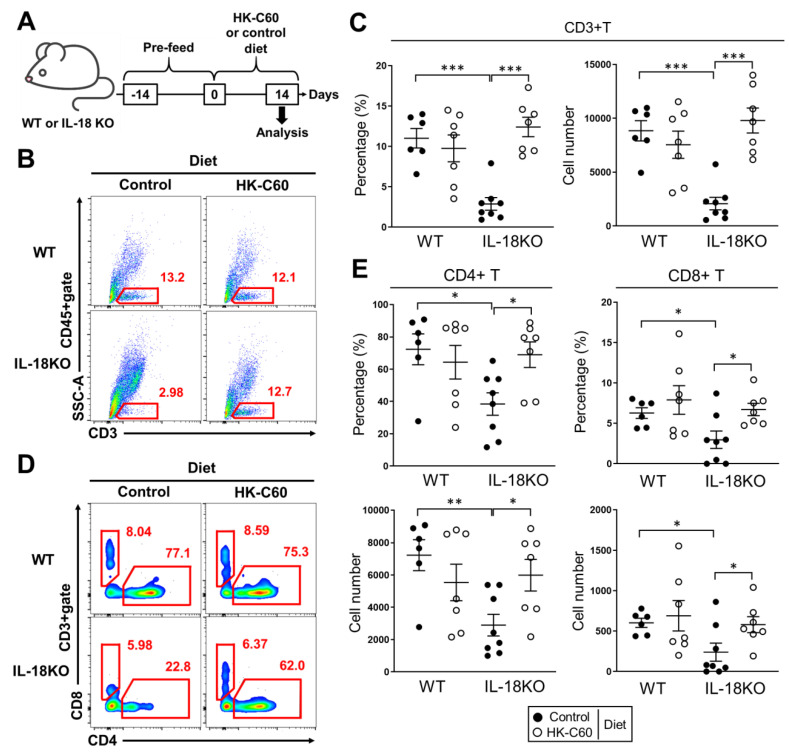
T cell population which specifically decreased in small intestinal lamina propria (SI-LP) is recovered by HK-C60 diet in aged IL-18KO mice. (**A**) The design of diet in WT and IL-18O aged mice. The mice were fed with control diet for 2 weeks for habituation, then were further fed with HK-C60 or control diet for 2 weeks. After the diet, the mice were used for analysis. (**B**–**E**) The Characterization of T cell population in SI-LP. The isolated SI-LP cells were analyzed by flow cytometry. The representative image of CD3+ T cells in CD45+ gate (**B**), CD4+ and CD8+ T cells in CD45+CD3+ gate (**D**). The percentage of CD3+ T cells in CD45+ gate (**C**, left), CD4+ and CD8+ T cells in CD45+CD3+ gate (**E**, upper). The cell number of CD3+ T cells in CD45+gate (per 10^5^) (**C**, right), CD4+ and CD8+ T cells in CD45+CD3+gate (per 10^4^) (**E**, lower). The data were shown as the representative or mean ± SEM of at least six samples in three independent experiments. Each dot indicates the data from one sample. A Mann–Whitney U test was used to analyze data for significant differences. Values of * *p* < 0.05, ** *p* < 0.01, and *** *p* < 0.001 were regarded as significant.

Since IL-18 has been identified as an important cytokine in T cell activation and maintenance in various tissues and organs [29], we investigated whether IL-18 deficiency influences in T cell population of small intestine or not. Firstly, we found a specifically decreased cell population in SI-LP of aged (over 10 months) IL-18KO mice as compared to that of aged WT mice (Supplementary Figure S1, red circle). As a major influenced population, we identified that CD3+ T cells were dramatically decreased in SI-LP of aged IL-18 KO (Figure 1B). Both the percentage and number of CD3+ T cells were significantly decreased in SI-LP of aged IL-18-KO mice compared with WT mice (Figure 1C). Interestingly, the decreasing of CD3+ T cells was age dependently accelerated event in SI-LP of IL-18KO mice. Both percentage and number of CD3+ T cells were greatly decreased in aged IL-18KO mice as compared to those of aged WT mice (Supplementary Figure S2). In fact, IL-18KO mice showed a mild decreasing of CD3+ T cell population in SI-LP in their young age (10–12 weeks) compared with same age of WT mice, while the grade of decreasing was much severe in the aged group (Supplementary Figure S2). On the other hand, WT mice didn’t show any significant change in the CD3+ T cell population between young and aged group.

*Lactococcus lactis* subsp. *Cremoris* C60 was identified from chase, and has already characterized as an inducer of cytokine production in BMDCs and primary splenocytes [30]. We also confirmed that HK-C60 induced cytokines production and cellular activation in BMDCs (Supplementary Figure S3). Since other strains in *Lactococcus lactis* subsp. *Cremoris* have already reported as available bacteria in probiotics [31,32], it was expected that C60 also possesses probiotic function especially in intestinal immunological environment. To investigate the immunological modificative effect of C60 in intestinal T cell environment especially in aged mice, we fed both WT and IL-18KO aged mice with control or HK-C60 diet for two weeks (Figure 1A). We found that the CD3+ T cell population, which almost disappeared in control diet, was completely recovered in the SI-LP of aged IL-18KO mice in HK-C60 diet (Figure 1B). The percentage and cell number of CD3+ T cells were significantly decreased in aged IL-18KO mice as compared to those of aged WT mice in control diet, while both of those were recovered in HK-C60 diet (Figure 1C). On the other hand, the CD3+ T cell population was sustained in SI-LP of aged WT mice between control and HK-C60 diet (Figure 1B,C). For further characterization in the CD3+ T cell population, we analyzed CD4+ and CD8+ T cells in SI-LP. The CD4+ T cell population was dramatically decreased in aged IL-18KO mice in the control diet. However, the population was recovered in the HK-C60 diet (Figure 1D). Both the percentage and number of CD4+ T cells in SI-LP were significantly decreased in aged IL-18KO mice compared with aged WT mice, while those were recovered to similar level as aged WT mice in HK-C60 diet (Figure 1E). In fact, CD8+ T cells were also influenced by IL-18-deficiency in aged mice, because the percentage and number were slightly decreased in SI-LP of aged IL-18KO mice as compared to those of WT (Figure 1D,E). However, it was much milder than the abnormality observed in the CD4+ T cell population in aged IL-18KO mice.

Since spleen and peripheral blood showed almost similar percentage and cell number of both CD4+ and CD8+ T cells between WT and IL-18KO aged mice, the decreasing of T cell population was SI-LP specific manner in aged IL-18KO mice (Supplementary Figure S4A,B). In addition, the population of CD4+ and CD8+ T cells in PPs were also similar between WT and IL-18KO aged mice, except a notable increasing of the percentage of CD4+ T cells in aged IL-18KO mice with HK-C60 diet as compared to that of aged WT mice (Supplementary Figure S4C). The thymic T cell development was also investigated in WT and IL-18KO aged mice. There was no difference between these two strains (Supplementary Figure S5).

Thus, CD3+ T cell population is selectively decreased in SI-LP of aged IL-18KO mice. In addition, CD4+ T cell is one of the most influenced subsets in the IL-18-deficient environment. HK-C60 diet possesses remarkable effect to recover the age-associated CD3+ T cells decreasing in aged IL-18KO mice.

### 3.2. HK-C60 Diet Increases IFN-γ Producing CD4+ T Cells in Small Intestine of Aged IL-18KO Mice

**Figure 2:**
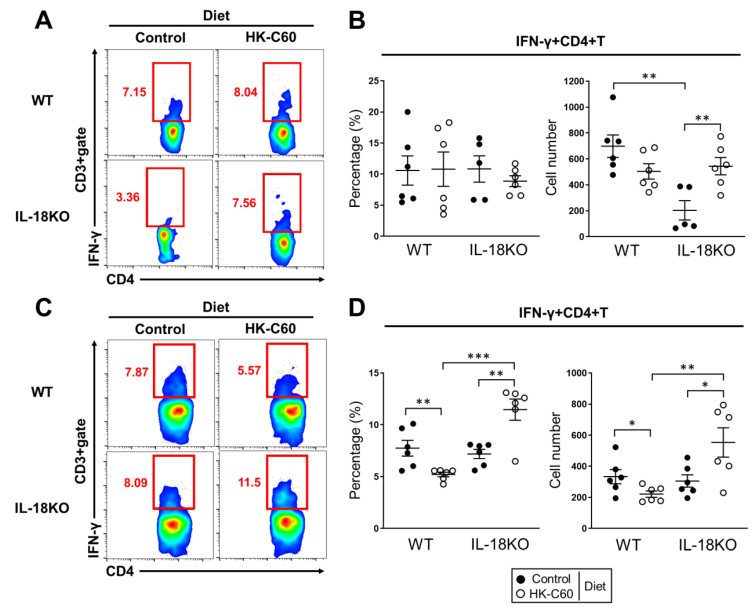
Th1 cell population was increased in small intestine of IL-18KO mice with HK-C60 diet. (**A**–**D**) Effector CD4+ T cell population in SI-LP and PPs isolated cells were analyzed by flow cytometry. The isolated cells were re-stimulated with PMA/Ionomycin combined with GolgiStop^TM^ at 37 °C for 5 h. The representative image of IFN-γ+CD4+ T (Th1) cell population in CD45+CD3+ gate in SI-LP (**A**) and PPs (**C**). The percentage of Th1 cells in CD45+CD3+CD4+ gate in SI-LP (**B**, left) and PPs (**D**, left). The number of Th1 cells in CD45+CD3+gate (per 10^4^) in SI-LP (**B**, right) and PPs (**D**, right). The data were shown as the representative or mean ± SEM of six samples in three independent experiments. Each dot indicates the data from one sample. A Mann–Whitney U test was used to analyze data for significant differences. Values of * *p* < 0.05, ** *p* < 0.01, and *** *p* < 0.001 were regarded as significant.

As same as skin and mucosa, effector CD4+ T cells are indispensable player to establish healthy immune responses in small intestine [33]. We next investigated the effector CD4+ T cell population in SI-LP of aged mice. IFN-γ+CD4+ T (Th1) cells in SI-LP were dramatically decreased in aged IL-18KO mice as compared to that of aged WT mice in control diet (Figure 2A, left). However, the Th1 cell population was completely recovered in the aged IL-18KO mice fed with HK-C60 diet (Figure 2A, right). The number of Th1 cells in SI-LP was significantly decreased in aged IL-18KO mice fed with control diet as compared to that of aged WT mice. While the cell number was recovered in aged IL-18KO mice with HK-C60 diet, and the value was reached to the similar level as aged WT mice (Figure 2B).

Since PP is a primary lymphoid tissue to provide immune cells to LP or epithelium in small intestine [34], we investigated Th1 cell population in PPs isolated from the aged mice fed with HK-C60 or control diet. The percentage and number of Th1 cells were no difference between WT and IL-18KO aged mice in control diet, while the values were significantly increased in aged IL-18KO mice fed with HK-C60 diet as compared to that of aged WT mice (Figure 2C,D).

Thus, HK-C60 diet restores small intestinal Th1 cell population in age IL-18KO mice.

### 3.3. HK-C60 Enhances Cellular Activity of CD4+ T Cells in PP of IL-18KO Mice

**Figure 3:**
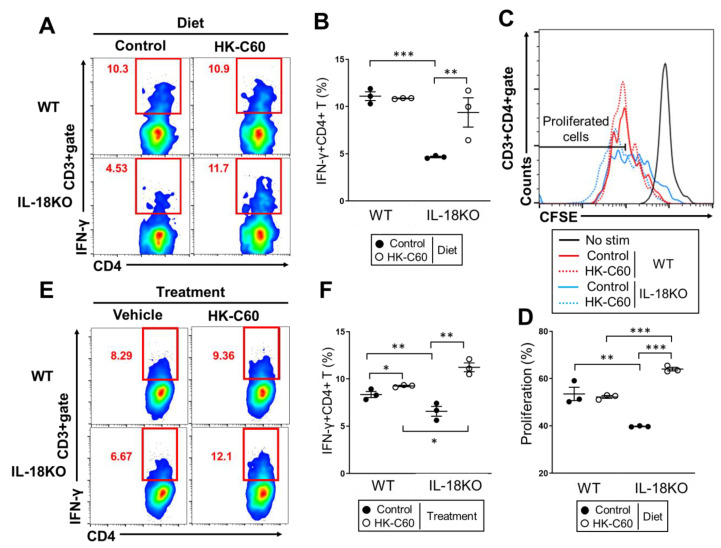
TCR-dependent CD4+ T cells activity is enhanced in IL-18-deficient PP by exposing with HK-C60. (**A**–**D**) In vitro stimulation of PP isolated cells. The cells were isolated from PPs of aged mice fed with HK-C60 or control diet. The cells were stimulated with anti-CD3/CD28 mAb at 37 °C for 48 h. (**A**,**B**) IFN-γ+CD4+ T (Th1) cell population in the culture. The cultured PP cells were re-stimulated with PMA/Ionomycin combined with GolgiStop^TM^ at the last 5 h of culture. The representative image and percentage of Th1 cell population in CD3+gate are shown as (**A**,**B**) respectively. (**C**,**D**) The proliferation of CD4+ T cells in the culture. The cells were stained with CFSE prior seeding on the plate. The proliferated CD4+ T cells were analyzed by flow cytometry. The representative image of CD4+ T cells proliferation and the percentage of proliferated cell in CD3+CD4+gate are shown as (**C**,**D**) respectively. (**E**,**F**) Ex vivo stimulation of PP isolated cells. The cells were isolated from PPs of aged mice with normal diet. The cells were stimulated with anti-CD3/CD28 mAb and were further treated with HK-C60 or vehicle control. The culture was incubated at 37 °C for 48 h. At the last 5h, the cells were re-stimulated with PMA/Ionomycin combined with GolgiStop^TM^. The IFN-γ+CD4+ T (Th1) cells were analyzed by flow cytometry. The representative image and percentage of Th1 cell population in CD3+gate are shown as (**E**,**F**) respectively. The data were shown as the representative or mean ± SEM of six samples in three independent experiments. Each dot indicates the data from one sample. A Mann–Whitney U test was used to analyze data for significant differences. Values of * *p* < 0.05, ** *p* < 0.01, and *** *p* < 0.001 were regarded as significant.

We next investigated how CD4+ T cells function was upregulated by HK-C60 diet in small intestine. Since the increasing of Th1 cells was observed in PPs, and the total number of PP isolated cells were increased by HK-C60 diet in both WT and IL-18KO mice (Figure 2C,D, Supplementary Figure S6), we investigated CD4+ T cells activity in PPs which is one of the susceptible lymphoid tissues of small intestine in the diet [35]. The CD4+ T cells activity, especially for TCR-restricted manner, was analyzed by in vitro stimulation with anti-CD3/CD28 mAb treatment. The Th1 cells activity was significantly attenuated in aged IL-18KO mice with control diet as compare to that of aged WT mice (Figure 3A,B).On the other hand, the activity was enhanced in aged IL-18KO mice fed with HK-C60 diet. The percentage of Th1 cells was significantly increased in the IL-18KO mice culture originated from HK-C60 diet as compared to that of control diet. The value was reached to that of aged WT mice (Figure 3A,B). In addition, the cell proliferation of PP CD4+ T cells were significantly attenuated in the culture originated from aged IL-18KO mice as compared to that of WT mice in control diet. On the other hand, the cell proliferation was promoted in the IL-18-deficient culture prepared from the mice with HK-C60 diet. The proliferation rate of CD4+ T cells was significantly higher than that of WT culture (Figure 3C,D).

**Figure 4:**
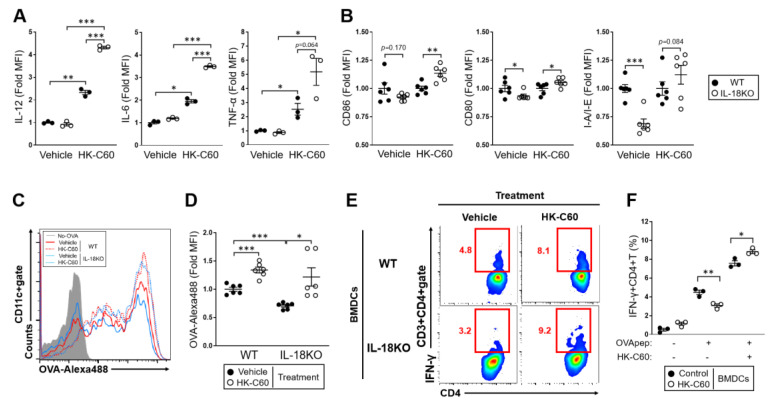
The function of IL-18-deficient DCs is enhanced by HK-C60 stimulation. (**A**) Intracellular cytokine detection in HK-C60 stimulated BMDCs. The BMDCs were treated with HK-C60 or vehicle control at 37 °C for 6 h in the presence of GolgiStop^TM^. The cytokine expressions in CD11c+ gate were analyzed by flow cytometry. The MFIs were calculated from the analysis. (**B**) Cellular activation of HK-C60 stimulated BMDCs. The BMDCs were treated with HK-C60 or vehicle control at 37 °C for 24 h. The expression of CD80, CD86 and I-A/I-E in CD11c+ gate were analyzed by flow cytometry. The MFIs were calculated from the analysis. (**C**,**D**) Antigen uptake assay in BMDCs. BMDCs were co-incubated with OVA-Alexa 488 with or without HK-C60. The representative image of OVA uptake in flow cytometry (**C**) and the MFIs indicated the signal from uptaken OVA-Alexa 488 (**D**) in the analysis. (**E**,**F**) Antigen presentation assay to CD4+ T cells. BMDCs were cocultured with DO11.10 CD4+ T cells (1:5) in the presence of OVA_323–339_ peptide with or without HK-C60 stimulation at 37 °C for 72 h. At the last 5 h, the cells were re-stimulated with PMA/Ionomycin combined with GolgiStop^TM^. The IFN-γ+CD4+ T (Th1) cells were analyzed by flow cytometry. The representative image of Th1 cell population (**E**) and the percentage of Th1 cells (**F**) in CD3+CD4+ gate in the analysis. The data were shown as the representative or mean ± SEM of at least three samples in three independent experiments. Each dot indicates the data from one sample. A Mann–Whitney U test was used to analyze data for significant differences. Values of * *p* < 0.05, ** *p* < 0.01, and *** *p* < 0.001 were regarded as significant.

To investigate the influence of HK-C60 exposure in TCR-dependent activity of CD4+ T cells, we performed ex vivo stimulation assay which mimic natural internal environment of PP [5]. The PP isolated cells were cultured on the plate with anti-CD3/CD28 mAb treatment combined with or without HK-C60 stimulation. The IL-18-deficient culture showed a smaller percentage of Th1 cells than WT culture in vehicle control treatment (Figure 3E left,F). However, the IL-18-deficient culture showed a significant increase of Th1 cells percentage with HK-C60 stimulation as compared to that of WT culture (Figure 4E left,F). The HK-C60 mediated functional upregulation of CD4+ T cells wasn’t confirmed in the culture with only CD4+ T cells (unpublished data). This evidence implied that the upregulation of CD4+ T cells function required the whole internal environment of PP in HK-C60 stimulation.

Taken together, HK-C60 promotes CD4+ T cells activity such as IFN-γ production and cell proliferation, which are induced by TCR-dependent stimulation, in PP of IL-18KO mice.

### 3.4. The IL-18-Deficient DCs Function Is over Activated by HK-C60 Stimulation, Which Directory Influences in Th1 Cells Generation

In the small intestine, DCs work for the generation and activation of effector CD4+ T cells through their antigen presentation activity, and regulate the immunological environment by producing cytokines [36,37]. Intestinal DCs are frequently exposed with various food derived stimulators which orally up taken then transferred to gut [38]. It is well known that LAB stimulate DCs and regulate their function, which directly influences the T cells character in the small intestine [39]. To investigate the immunological influence of HK-C60 in DCs function, we performed a BMDCs stimulation assay with HK-C60. The cytokine production, such as IL-12, IL-6 and TNF-α were all greatly induced by HK-C60 stimulation in both WT and IL-18-deficient BMDCs. The upregulation level of these cytokines production in IL-18-deficient cells was significantly higher than WT cells (Figure 4A). The cellular activation was also investigated in both WT and IL-18-deficient BMDCs. As a basal status, the expression level of CD86, CD80 and MHC class II (I-A/I-E) in WT BMDCs were all higher than those of IL-18-deficient cells. I-A/I-E expression was dramatically downregulated in IL-18-deficient BMDCs (Figure 4B). Primary PP DCs also showed the down regulation of these surface molecules expression in IL-18KO mice compared with WT mice (Supplementary Figure S7). In HK-C60 stimulation, IL-18-deficient BMDCs showed overactivation, which was characterized by the significant upregulation of CD86, CD80, and I-A/I-E expression as compared to that of WT cells (Figure 4B).

To investigate the DCs function related with antigen presentation into CD4+ T cells activation, we performed both antigen uptake assay and antigen presentation assay. The BMDCs showed an abundant uptake of OVA as an exogenous antigen, and the activity in WT cells was significantly higher than IL-18-deficient BMDCs in the vehicle treatment. The OVA uptaking was enhanced in both WT and IL-18-deficient BMDCs in HK-C60 stimulation compared with vehicle control, while the level in IL-18-deficient cells was reached to almost similar as that of WT cells (Figure 4C,D).

In the antigen presentation assay, WT or IL-18-deficient BMDCs were co-cultured with splenic CD4+ T cells isolated from DO11.10 mice in the presence of OVA_323–339_ peptide as an antigen. Furthermore, some of the cultures were treated with HK-C60. The percentage of Th1 cells, which were generated with antigen specific manner, in IL-18KO BMDCs culture was smaller than that of WT culture (Figure 4E left,F). In the presence of HK-C60, both WT and IL-18-deficient BMDCs cultures showed an enhancement of Th1 cells generation. However, the Th1 cell population was significantly increased in IL-18-deficient BMDCs culture as compared to that of WT cells culture in the HK-C60 stimulation (Figure 4E right,F).

Taken together, HK-C60 stimulation induces over activation in IL-18-deficient DCs which leads to enhancing the immunological function in the cells. In addition, the HK-C60 mediated DCs activation is directory reflected in the upregulation of effector CD4+ T cells generation in IL-18-deficient environment.

## 4. Discussion

The intestinal immunological environment has been focused on as an important factor in the establishment of natural defense against various infectious diseases and immunological disorders [40,41]. Recently, preventive medicine has been gradually recognized as an important concept in our heath. As one of the contributing approaches, probiotics has been interested in the establishment of a healthy intestinal immunological environment. Especially for age-associated immunological alteration in the intestine, the environment frequently fails to induce a healthy immune response [42]. In this report, we showed that *Lactococcus lactis* subsp. *Cremoris* C60 possessed an ability to restore or maintain T cells activity in the small intestine of aged IL-18-deficient mice. The CD3+ T cell population, which completely disappeared in SI-LP, was dramatically recovered in aged IL-18KO mice in HK-C60 diet (Figure 1). In addition, the CD4+ T cells function was also restored in the small intestine of IL-18KO mice fed with a HK-C60 diet. We found the significant upregulation of IFN-γ production and cell proliferation in CD4+ T cells as compared to the mice in control diet (Figure 2 and Figure 3A–D). This is the first report that HK-LAB diet directly regulates CD4+ T cells activity in small intestine of aged IL-18KO mice.

As a mechanism in the functional upregulation of intestinal CD4+ T cells in HK-C60 diet, we found that DCs might have played an important role. HK-C60 stimulation enhanced both cytokine production and cellular activation those of which attenuated in the basal level of IL-18KO deficient BMDCs compared with WT cells (Figure 4A,B). In addition, the HK-C60 stimulation enhanced antigen uptake and antigen presentation in IL-18-deficient BMDCs which directly contributed into Th1 cells generation with antigen specific manner (Figure 4C–F). Furthermore, ex vivo stimulation of IL-18-deficient PP isolated cells (whole isolated cells containing DCs) showed that IFN-γ production in TCR-dependently stimulated CD4+ T cells were over upregulated in the presence of HK-C60, and the level was significantly higher than that of WT culture (Figure 3E,F). This result implied that the whole internal environment, including DCs contribution, of PP was necessary to induce the upregulation of CD4+ T cells activity in HK-C60 stimulation. On the other hand, the culture of only IL-18-deficient CD4+ T cells didn’t show any upregulation of cytokine production or differentiation into specific subset in HK-C60 stimulation as the same result as WT CD4+ T cells (unpublished data). This evidence also implied that HK-C60 induces the alteration of CD4+ T cells activity through other immune cells such as DCs. These findings provide strong evidence that probiotic LAB possess an immunomodulatory effect in the intestinal immune system by controlling the tight connection between innate and adaptive immune response.

We found that IL-18-deficiency influences into intestinal T cells environment in this study. It was a novel finding that the dramatic decreasing of T cell population in SI-LP of aged IL-18KO mice (Figure 1B,C and Supplementary Figure S2). The recovery of CD4+ T cells which disappeared in SI-LP of aged IL-18KO mice was also an unexpectedly dramatic event in HK-C60 diet (Figure 1D,E). In addition, we found that the upregulation of CD4+ T cells function in aged IL-18KO mice was HK-C60 dependent manner which was confirmed by both in vivo and in vitro experiments (Figure 2 and Figure 3). In fact, the critical impairment of the CD4+ T cells function was thought as an age-associated event in IL-18KO mice, because the decreasing of CD3+ T cells was age-dependently accelerated in IL-18KO mice (Supplementary Figure S2). We have never found a clue to reveal the exact mechanism, except the correlation with DCs function, in the age-associated T cells functional failure in IL-18KO mice. Further study must be required in this point by focusing on T cell itself as well as on other immune cells. As a non-immunological response, we must consider the NLRs mediated response in the IL-18 production from epithelial cells [22]. Recently, NLRs have been reported as receptors for the recognition of probiotic bacteria including LAB in the epithelial cells [43,44]. We still have to gain clear evidence to fully explain the immunomodulatory effect of HK-C60 in small intestine. However, we remain sure that a HK-LAB diet has a strong potential to improve the intestinal immunological environment which is impaired by ageing process.

The exact mechanism of the DCs activation in HK-C60 stimulation must be revealed by future detail study. In fact, we have already found that BMDCs prepared from myeloid differentiation primary response 88 (MyD88)-KO mice, which don’t respond to TLRs ligand stimulation except against TLR3 [45], failed to produce IL-6, IL-12, and TNF-α in HK-C60 stimulation (unpublished data). Furthermore, WT BMDCs with TLR2 blocking by using mAb suppressed these cytokines production in HK-C60 stimulation (unpublished data). Thus, the TLRs–MyD88 axis may have responsibility in the recognition of HK-C60 and induction of the activation signal in DCs. Some studies have already revealed that the immune cells sensed and captured LAB through TLRs [8,9,46], so this evidence strongly supports our preliminary finding and hypothesis in the mechanism of HK-C60 mediated DCs activation.

## 5. Conclusions

**Figure 5:**
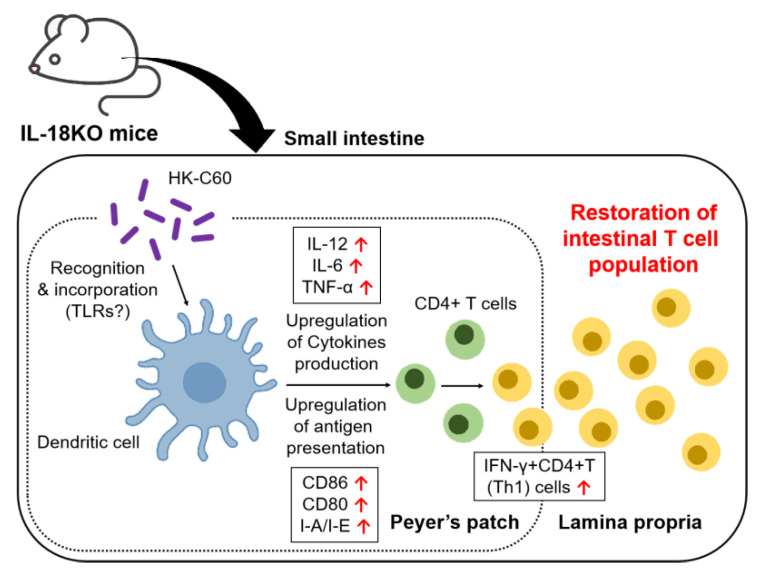
The hypothetical model of immunomodulatory effect of HK-C60 in DCs based effector CD4+ T cells generation in small intestine of IL-18KO mice. HK-C60 stimulation induces over activation of IL-18-deficinet DCs. The DCs activity directory correlated with antigen specific effector CD4+ T cells generation, such as cytokine production and expression of antigen presenting molecules as well as antigen uptake, are all upregulated in the HK-C60 stimulated IL-18-deficient DCs. As a result, effector CD4+ T cells, such as Th1 cells, generation is promoted by the over activated IL-18-deficient DCs.

The T cell population is specifically decreased in the SI-LP of IL-18KO mice. As the most influenced subset in the T cell lineage, the CD4+ T cell population is also decreased in the SI-LP of IL-18KO mice. In addition, the function of effector CD4+ T cells, such as Th1 cells, is attenuated in the IL-18-deficient small intestine. HK-C60 diet restores T cell population including CD4+ T cells with functional upregulation in SI-LP of IL-18KO mice. The generation Th1 cells, which is suppressed in IL-18-deficient small intestine, is promoted in the HK-C60 diet. As a possible mechanism in the T cell restoring process, the upregulation of DCs function is important in the small intestine of IL-18KO mice. DCs are over activated by exposing with HK-C60 characterized by an increase of cytokines production and upregulation of surface molecules expression, as well as antigen uptake, which is tightly correlated with antigen presenting activity. Finally, these upregulated activities totally promote antigen presentation to CD4+ T cells, and enhance effector CD4+ T cells generation in the small intestine of IL-18-deficient (Figure 5).

## Supplementary Materials

The following are available online at https://www.mdpi.com/2072-6643/12/11/3287/s1, Table S1. The Ab staining combination in flow cytometry analysis; Figure S1: The decreasing of specific population in CD45+ cells in SI-LP of IL-18KO mice, Figure S2: Age-associate decreasing of CD3+ T cells in SI-LP, Figure S3: HK-C60 induces cytokine production and cellular activation in BMDCs, Figure S4: The characterization of T cells in non-intestinal environment and PP, Figure S5: The thymic development of T cells, Figure S6: The number of PP isolated cells, Figure S7: The basal status of primary PP DCs.
