# Supplementary material for: Lactococcus lactis subsp. Cremoris C60 restores T Cell Population in Small Intestinal Lamina Propria in Aged Interleukin-18 Deficient Mice
Source: Nutrients. 2020 Oct 27;12(11):3287. doi: 10.3390/nu12113287 (PMC7693701; doi:10.3390/nu12113287)
Supplement: Supplementary file 1 [file nutrients-12-03287-s001.pdf]

Supplemental materials

T cells
(primary cells prepared from SI-LP, PP, peripheral blood and spleen)

Antigen	Fluorochrome	Clone	Concentration (μg/mL)
Anti-CD45	FITC	30-F11	0.25
Anti-CD3ε	APC	17A2	0.25
Anti-CD4	APC/Cy7	GK1.5	0.5
Anti-CD8α	Pacific Blue	53-6.7	0.25
Anti-IFN-γ	PE	XMG1.2	0.5

DCs (primary cells prepared from PP)

Antigen	Fluorochrome	Clone	Concentration (μg/mL)
Anti-CD45	PE/Cy7	30-F11	0.25
CD11c	BV421	N418	0.5
Anti-CD80	FITC	16-10A1	0.25
Anti-CD86	PE	GL-1	0.25
Anti-HMCII	APC	M5/114.15.2	0.25

T cells
(in vitro and ex vivo stimulated)

Antigen	Fluorochrome	Clone	Concentration (μg/mL)
Anti-CD45	FITC	30-F11	0.25
Anti-CD3ε	APC	17A2	0.25
Anti-CD4	BV421	GK1.5	0.5
Anti-CD8α	PE/Cy7	53-6.7	0.25
Anti-IFN-γ	PE	XMG1.2	0.5

DCs (in vitro stimulation)

Antigen	Fluorochrome	Clone	Concentration (μg/mL)
Anti-CD11c	BV421	N418	0.5
Anti-CD80	FITC	16-10A1	0.25
Anti-CD86	PE	GL-1	0.25
Anti-HMCII	APC	M5/114.15.2	0.25

Table S1. The Ab staining combination in flow cytometry analysis

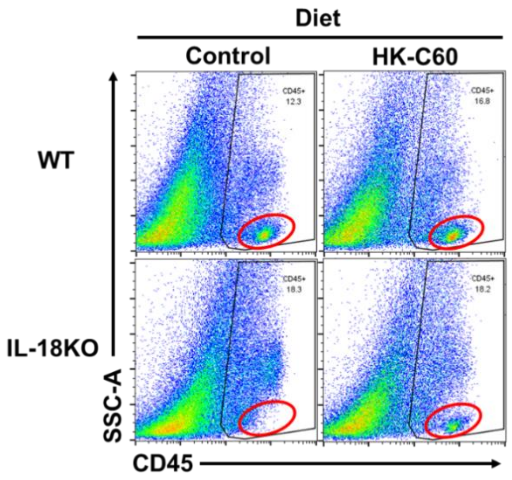

Figure S1. The decreasing of specific population in CD45+ gated cells in SI-LP of IL-18KO mice The SI-LP cells were isolated from small intestine of aged WT and IL-18KO mice with HK-C60 or control diet. The cells were analyzed by flow cytometry. The red circle indicates lymphoid cell population in CD45+ gate which specifically disappeared in SI-LP of IL-18KO mice in control diet. The data were shown as the representative of eight samples in each group in three independent analyses.

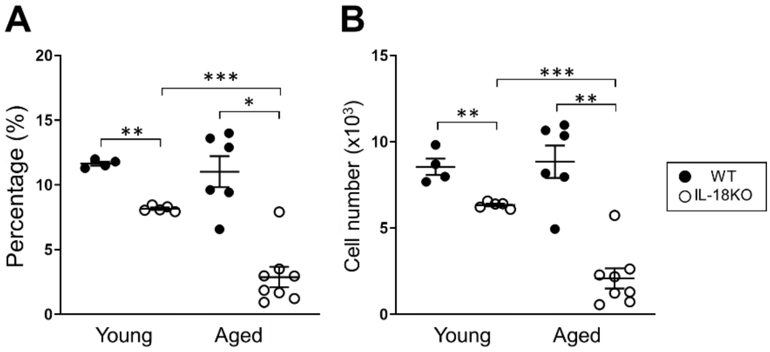

Figure S2. Age-associate decreasing of CD3+ T cells in SI-LP

The SI-LP cells were isolated from small intestine of both WT and IL-18KO mice with normal diet. The percentage (in CD45+ gate) (A) and number (per 10^5 of CD45+ cells) (B) of CD3+ T cells were analyzed by flow cytometry. The data were shown as the mean ± SEM of five (young; 10-12 weeks) or six (aged; over10 months) samples in three independent analyses. Each dot indicates the data from one sample. A Mann–Whitney U test was used to analyze data for significant differences. Values of **p*<0.05, ***p*<0.01, and ****p*<0.001 were regarded as significant.

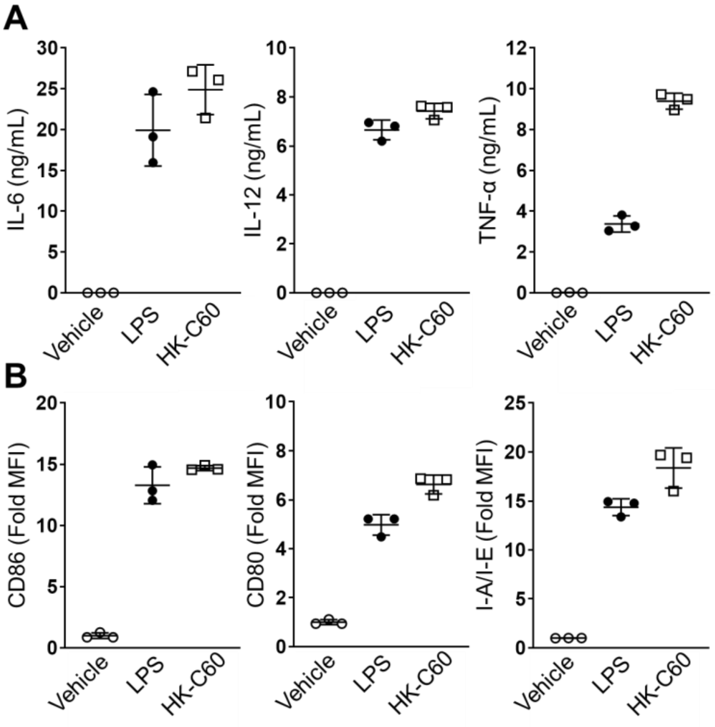

Figure S3. HK-C60 induces cytokine production and cellular activation in BMDCs

A-B) The BMDCs were stimulated with LPS (10 ng/mL) or HK-C60. Some cultures were treated with vehicle control. The cultures were incubated at 37°C for 24 h, then cytokine production was

measured by ELISA (A). The expression of CD86, CD80 and I-A/I-E were analyzed by flow cytometry (B). The data were shown as the mean ± SEM of three samples in three independent analyses. Each dot indicates the data from one sample.

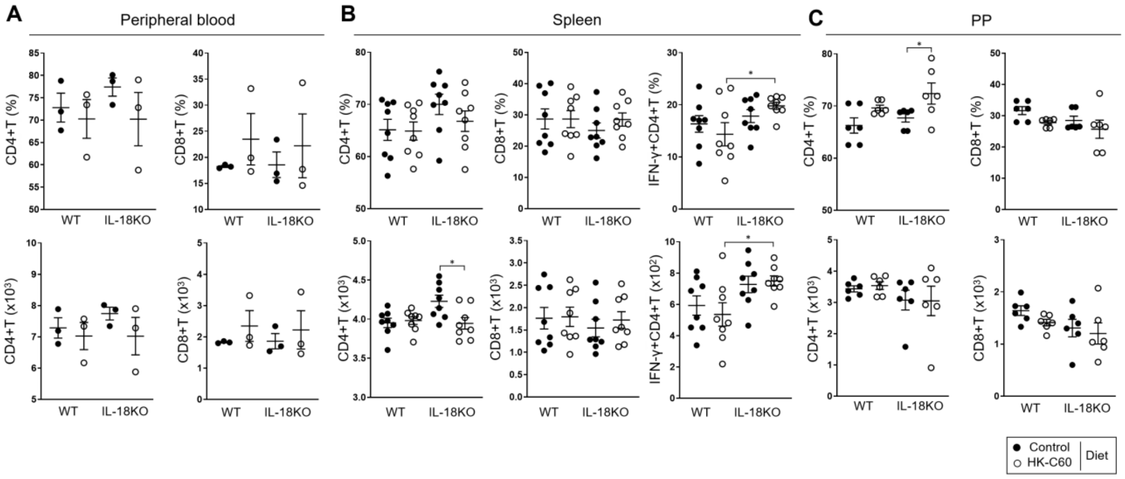

Figure S4. The characterization of T cells in non-intestinal environment and PP

A-C) Peripheral blood, spleen and PPs were gained from both WT and IL-18KO aged mice with control or HK-C60 diet. The cells were prepared from each sample, then the T cells were analyzed by flow cytometry. For Th1 cells analysis, the cells were re-stimulated with PMA/ionomycin combined with GolgiStop™ at 37℃ for 5h prior the analysis. The percentage (in CD45+CD3+ gate) and number (per 10^5 of CD3+ T cells) of CD4+ and CD8+ T cells in peripheral blood (A), spleen (B) and PP (C). The percentage and number of Th1 cells in spleen (C). The data were shown as the mean ± SEM of at least three samples in three independent analyses. Each dot indicates the data from one sample. A Mann–Whitney U test was used to analyze data for significant differences. Values of *p<0.05 was regarded as significant.

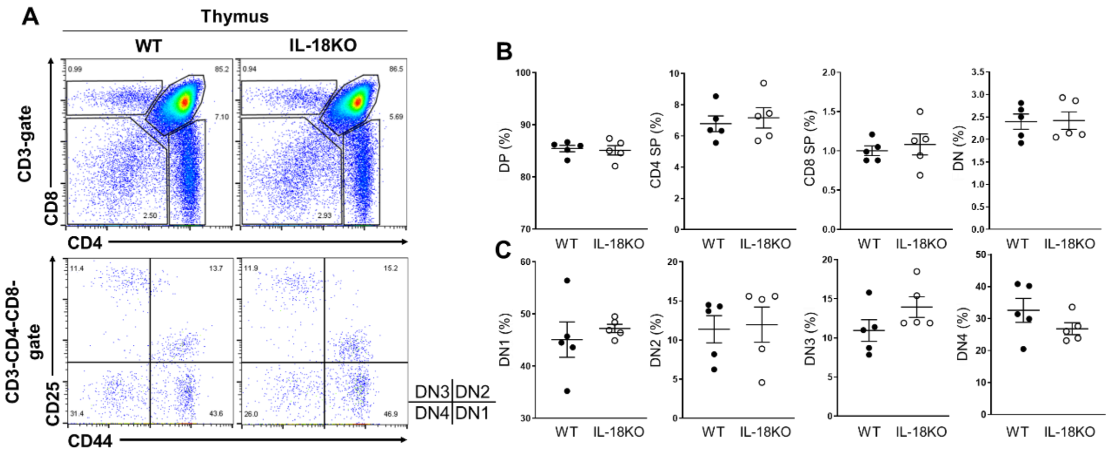

Figure S5. The thymic development of T cells

Thymocytes were analyzed by flow cytometry to investigate T cell development. A) Representative image of CD4/CD8 staining in CD3- gate and CD44/CD25 staining in CD3-CD4-CD8- gate. B) The percentage of CD4/CD8 double positive (DP), CD4 single positive (CD4SP), CD8 single positive (CD8SP) and double negative (DN) in CD3- gate. C) The percentage of CD44+CD25- (DN1), CD44+CD25+ (DN2), CD44-CD25+ (DN3) and CD44-CD25- (DN4) in CD3-CD4-CD8- gate. The data were shown as the representative and mean ± SEM of five samples in three independent analyses. Each dot indicates the data from one sample.

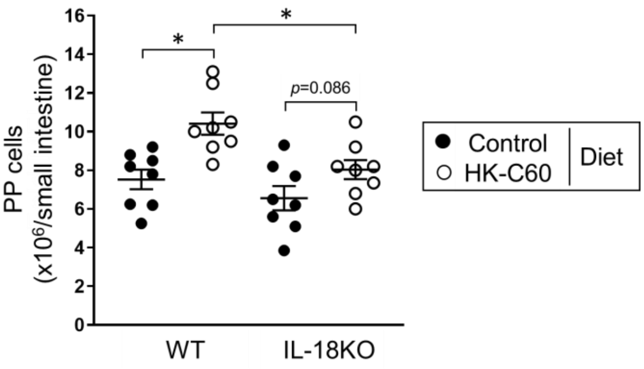

Figure S6. The number of PP isolated cells

PPs were extracted from small intestine of both aged WT and IL-18KO mice with control or HK-C60 diet. The total number of PP isolated cells was counted in each mouse. The data were shown as the mean ± SEM of eight samples in three independent analyses. Each dot indicates the data from one sample. A Mann–Whitney U test was used to analyze data for significant differences. Values of *p<0.01 was regarded as significant.

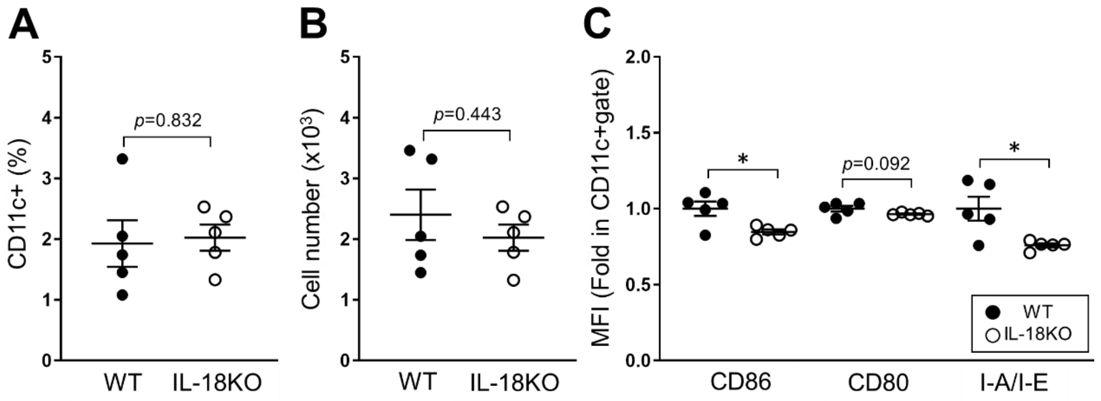

Figure S7. The basal status of primary PP DCs

A-C) The PPs were extracted from small intestine of both aged WT and aged IL-18KO mice with normal diet. The cells were isolated form the PPs, then the percentage (A), number (B) and surface markers expression (CD80, CD86 and I-A/I-E) of CD11c+ DCs in CD45+ gate were analyzed by flow cytometry. The data were shown as the mean ± SEM of five samples in three independent analyses. Each dot indicates the data from one sample. A Mann–Whitney U test was used to analyze data for significant differences. Values of *p<0.05 was regarded as significant.
